# Progress of fluorescence imaging in lymph node dissection surgery for prostate and bladder cancer

**DOI:** 10.3389/fonc.2024.1395284

**Published:** 2024-10-04

**Authors:** Mingquan Xu, Panpan Li, Jinzheng Wei, Pengyu Yan, Yunmeng Zhang, Xinyu Guo, Chao Liu, Xiaofeng Yang

**Affiliations:** ^1^ Department of Urology, First Hospital of Shanxi Medical University, Taiyuan, ;China; ^2^ First Clinical Medical College, Shanxi Medical University, Taiyuan, ;China; ^3^ Department of Neurosurgery, First Hospital of Shanxi Medical University, Taiyuan, ;China; ^4^ Department of Orthopedics, First Hospital of Shanxi Medical University, Taiyuan, ;China

**Keywords:** fluorescence imaging, fluorescent contrast agent, pelvic lymph node dissection, lymph node metastasis, multimodal imaging

## Abstract

Fluorescence imaging is a relatively new imaging method used to visualize different tissue structures to help guide intraoperative operations, which has potential advantages with high sensitivity and contrast compared to conventional imaging. In this work, we review fluorescent contrast agents and devices used for lymphatic system imaging. Indocyanine green is the most widely utilized due to its high sensitivity, specificity, low background fluorescence, and safety profile. In prostate and bladder cancer lymph node dissection, the complex lymphatic drainage can result in missed metastatic nodes and extensive dissection increases the risk of complications like lymphocele, presenting a significant challenge for urologists. Fluorescence-guided sentinel lymph node dissection facilitates precise tumor staging. The combination of fluorescence and radiographic imaging improves the accuracy of lymph node staging. Multimodal imaging presents new potential for precisely identifying metastatic pelvic lymph nodes.

## 1 Introduction

Fluorescence imaging is a relatively new imaging method to visualize different types of tissues by fluorescence (1). It has many advantages over conventional imaging, such as real-time intraoperative imaging, enhanced contrast between tumor and surrounding tissues, identification of tiny occult lesions, and reduction of positive margins (2, 3). The key to fluorescence imaging lies in selecting appropriate fluorescent contrast agents and imaging systems (4). There is increasing interest in developing novel fluorescent contrast agents and fluorescent imaging systems and their use in imaging the lymphatic system (5). As early as 1999, Reynolds JS et al. demonstrated the potential of the fluorescent dye indocyanine green (ICG) for detecting spontaneous breast cancer and regional lymph nodes through animal experiments (6). In 2004, Nimura H et al. used ICG for sentinel lymph node (SLN) detection in gastric cancer and combined it with infrared ray electronic endoscopy to avoid missing metastatic lymph nodes (7). For lung cancer patients, fluorescence-guided SLN resection is also feasible (8). In 2007, a Japanese research team reported for the first time the use of ICG fluorescence lymphography for the diagnosis of lower limb lymphedema as an alternative to conventional lymphography (9). Additionally, ICG fluorescence imaging can be used to quantify lymphatic flow rate and pulse frequency, allowing for real-time, non-invasive monitoring of lymphatic function (10). We focus on the application of fluorescence imaging in lymph node dissection surgery for prostate and bladder cancer. Fluorescence imaging is used to visualize the SLN to improve traditional lymph node dissection procedures, enable more accurate surgical guidance, reduce surgical complications, and potentially improve patient prognosis.

Prostate cancer is the most common malignant tumor of the urinary system, with approximately 1.4 million new cases and 375,000 deaths per year worldwide. Bladder cancer is the second most common malignant tumor in the urinary system, with approximately 573,000 new cases and 213,000 deaths annually (11). Tumor metastasis is the main cause of patient death, and lymph node metastasis is an important route of tumor metastasis (12). About one-third of metastases in male malignant tumors first occur in the lymph nodes and are strongly associated with poor prognosis (13). The incidence of lymph node metastasis ranges from 5% to 70% in localized prostate cancer and from 16% to 35% in bladder cancer (14–16). For patients with bladder cancer in the presence of lymph node metastases, the 5-year survival rate is significantly lower, at about 35%. Surgical removal of tumor-associated lymph nodes can help to improve the prognosis, so pelvic lymph node dissection (PLND) is essential for patients with prostate and bladder cancers at risk of metastasis (17). However, PLND is a complex procedure and is prone to miss metastatic lymph nodes. It is not clear how many and how extensive lymph nodes should be removed for optimal patient benefit. Fluoroscopic SLN-guided lymph node dissection may reduce unnecessary extended dissection while avoiding missing metastatic lymph nodes beyond the extent of the dissection template. However, fluorescence imaging has a limited depth of detection, and combined with radionuclide imaging may overcome this drawback. In addition, various lymph node imaging techniques continue to advance, and the application of multimodal imaging with two or more imaging modalities allows for a more accurate assessment of lymph node status.

In this review, we first provide an overview of fluorescent contrast agents approved for clinical use or effective in clinical trials for bladder or prostate cancer. We summarize the current status of lymph node dissection for these cancers and point out the problems. Second, using the SLN technique, we analyze the current status of fluorescence imaging-guided lymph node dissection for prostate and bladder cancer. Third, other pelvic lymph node imaging techniques are summarized, including fluorescence combined with radionuclide imaging and multimodal imaging. Finally, the limitations of fluorescence imaging are summarized and its future development is prospected.

## 2 Fluorescence imaging

To obtain clear, high-resolution images for intraoperative guidance, researchers have introduced fluorescence imaging. Fluorescence imaging is an intraoperative optical imaging technique in which the operator uses an external light source to illuminate the tissue of interest, which excites a fluorescent contrast agent and identifies the target tissue by the fluorescence it emits. The technique is based on the Stokes shift principle: there is an energy difference between the absorption and emission spectra of the contrast agent, which is excited by an external light source and captured by a sensor, and displayed on a monitor. Compared with conventional imaging, fluorescence imaging enhances tumor-surrounding tissue contrast, reduces positive margins, identifies occult lesions, and improves routine surgical accuracy (2, 18). For prostate and bladder cancers, fluorescence imaging can assist lymph node dissection by obtaining high-resolution images of the lymph nodes, and the key to achieving good imaging lies in selecting appropriate fluorescent contrast agents and imaging systems. We provide experience in the further development and clinical translation of this technique by reviewing the fluorescent imaging agents and imaging systems.

Currently, the fluorescent imaging drugs that have obtained clinical approval in China include fluorescein sodium, methylene blue, ICG, and 5-aminolevulinic acid (5-ALA) (19). Among them, methylene blue, ICG, and 5-ALA can be utilized in surgical procedures for prostate or bladder cancer. Fluorescent probes used in clinical trials for prostate or bladder cancer also include hexaminolevulinate (HAL), hypericin, and IRDye800CW (**Table 1**) (1, 20). In addition, there are some natural fluorophores present in the human body that produce autofluorescence when excited by visible light, such as aromatic amino acids, riboflavin, and porphyrin (21).

**Table 1:** Optical characterization and clinical applications of the fluorescent agents.

Fluorescence Agent	Excitation/Emission (nm)	Route of Administration	Quantum Yield	Target	Lymphatic Imaging of Organs
**Fluorescein sodium**	490/530	Intravenous, subcutaneous, oral	0.76	Lymphatic/Vascular system; Cell imaging	Breast, lower extremities
**Indocyanine green**	780/820-830	Intravenous, local, subcutaneous, oral	0.14 (in water)	Lymphatic/Vascular/Urinary system; Tumor imaging	Prostate, bladder, breast, head and neck, lower extremities
**5-aminolevulinic acid and derivatives**	380-440/620	Intravesical, oral, topical, intravenous, subcutaneous	0.54 (PpIX)	Mitochondria in tumor cells, including bladder tumors	Bladder, head and neck
**Methylene blue**	670/690	Topical, intravenous, oral	0.52 (in water)	Lymphatic/nervous system; mitochondria	Prostate, breast, head and neck, colorectal
**IRDye800CW**	775/795	Intravenous, local	0.10 (in MeOH)	Binding to biomolecules to target tumors	Prostate, breast, head and neck, gastrointestinal tract, skin

Fluorescein sodium, one of the earliest fluorescent dyes discovered in the mid-19th century, is now widely used for glioma identification and ophthalmic angiography (Approved for use in most countries) (22). It can also be used for lymphatic imaging to show lymphatic drainage pathways (Off-label use) (23). An *in vivo* imaging study using the Firefly Fluorescence Imaging System showed that sodium fluorescein could be visualized alongside ICG in the same image (24)​. In lymphatic supermicrosurgery, sodium fluorescein assesses lymphatic drainage patency​ (25)​. In resource-limited settings, sodium fluorescein is an effective low-cost alternative to ICG (26)​. However, its emission spectrum overlaps with natural fluorophores, limiting its use.

The ideal fluorescence wavelength for surgery is located in the Near-infrared (NIR) region (650-900 nm), where light is less likely to be absorbed by tissues, penetrates deeper into tissues and is less affected by scattering effects and autofluorescence (27). A commonly used fluorescent dye in the NIR region is ICG (28). It is a water-soluble tricarbocyanine dye with a half-life of 3-4 minutes after intravenous injection, and it mainly binds to serum albumin and α-1 lipoprotein, is metabolized by the liver, and excreted through the bile (21). Intravenous injection of ICG can be used for breast cancer detection (29). Additionally, ICG combined with albumin in an appropriate ratio can enhance the fluorescence intensity and help in lymph node detection (30). Because of its high contrast and good resolution in fluorescence imaging, ICG is widely used in clinical surgery (Approved for use in most countries). In non-tumor surgery, it can be used for vascular imaging of the retina and liver function assessment. In the field of oncologic surgery, it can be used for real-time fluorescence angiography in laparoscopic colorectal cancer surgery to assess proximal and distal intestinal perfusion, thus reducing the occurrence of anastomotic fistula (31, 32). In addition, ICG allows rapid tracing of lymphatic drainage by intradermal, subcutaneous, or peritumoral injection. It identifies lymphatic flow from the breast to the axilla in real-time in breast cancer surgery, facilitating precise SLN resection. In prostatectomy, ICG can be used for visualizing prostate lymphatic drainage pathways (33, 34). For penile cancer, metastasis occurs first in the inguinal lymph nodes and then spreads to the pelvic lymph nodes, making inguinal lymphatic metastasis the most important prognostic indicator. The use of ICG fluorescence imaging improves the accuracy of lymph node identification in the inguinal region without increasing complications (35, 36). In 2017, ICG was demonstrated to be capable of emitting in the near-infrared region II window (NIR-II, 1000-1700 nm) fluorescence (37). In 2020, Hu et al. further validated the feasibility of intraoperative fluorescence imaging in the NIR-II region in a study of 23 patients with hepatocellular carcinoma (38). Fluorescence imaging in the NIR-II region has further improved in terms of spatial resolution, imaging contrast, and penetration depth (39–41).

5-ALA is a precursor substance of protoporphyrin IX (PpIX), which is processed in the heme synthesis pathway to produce PpIX with strong photosensitizing activity (42). Exogenous 5-ALA entering the human body can be selectively absorbed by tumor cells, accumulating excess PpIX within the cells, which fluoresces red and leads to tumor cell necrosis and apoptosis under light irradiation at specific wavelengths. Although 5-ALA does not belong to near-infrared light, 5-ALA and PpIX can naturally exist in the human body and thus have relatively low toxicity. Currently, 5-ALA is used for the photodynamic diagnosis of bladder cancer, improving the detection rate of carcinoma *in situ* (Approved for use in most countries) (43, 44). Some studies have also explored its use in determining surgical margins in prostate cancer (Off-label use) (45, 46). HAL, an ester derivative of 5-ALA, was approved by the Food and Drug Administration (FDA) in 2010 for use with blue-light cystoscopy to detect a wider range of Ta or T1 stage bladder lesions (47, 48).

Methylene blue is used for SLN localization in various cancers, including breast, cervical, penile, and malignant melanoma (Off-label use) (49). Its sensitivity for SLN detection in penile cancer was 56%, lower than ICG, and particularly poor in obese patients (50, 51). It can also be used as a photosensitizer in photodynamic therapy (Experimental use). It reduces the survival of prostate cancer cells (PC3) by inducing oxidative stress and autophagy (52). Additionally, it has significant cytotoxic effects on bladder cancer cells (MBT-2 cell line), mainly through damage to cell membranes and mitochondria​ (53). Its clinical potential is limited by low fluorescence yield and lack of functional groups for ligand addition (54). Additionally, methylene blue may cause severe allergic reactions, hypertension, skin necrosis, and rashes (55–57).

Hypericin acts as a fluorescent dye that induces apoptosis in cancer cells by generating reactive oxygen species through light excitation at specific wavelengths (58, 59). It has shown antitumor effects in several tumor models, especially bladder cancer (Experimental use) (60). The main drawback is its insolubility in water; it is only soluble in ethanol and 1% plasma protein solution. D’Hallewin et al. demonstrated that hypericin can be used for fluorescence detection of bladder cancer, with papillary tumors displaying a red fluorescence and a sensitivity of 93% for detecting carcinoma *in situ* (61). In a larger study of 87 patients, the sensitivity and specificity for detecting carcinoma *in situ* were 94% and 95%, respectively (62). Compared with 5-ALA, hypericin has a higher specificity for detecting carcinoma *in situ* and may reduce the number of biopsies. Therefore, hypericin plays an important role in the early diagnosis and surgical navigation of bladder cancer.

The wavelength of IRDye800CW is located in the NIR region, and its advantage lies in its ability to bind to a wide range of biomolecules such as peptides, antibodies, and other targeting vectors to form specifically targeted fluorescent contrast agents. Combining monoclonal antibodies targeting the epidermal growth factor receptor (cetuximab or panitumumab) with IRDye800CW enables SLN-specific visualization and precise identification of metastatic lymph nodes (63). This drug is FDA-approved for use in clinical trials only. Rosenthal et al.’s study of head and neck tumors showed that cetuximab-IRDye800CW compared to standard histopathological examination of 35 pathologically positive lymph nodes 34 (sensitivity 97.2%) were correctly identified and 401 of 435 negative lymph nodes (specificity 92.7%) were correctly assessed by fluoroscopic imaging, which could avoid unnecessary neck lymph node dissection (64). Panitumumab is the first fully humanized monoclonal antibody, and Panitumumab-IRDye800CW has high sensitivity (84.6%) and specificity (94.0%) in identifying metastatic lymph nodes and distinguishing metastatic lymph nodes of different sizes from benign lymph nodes (65). Prostate-specific membrane antigen (PSMA) is highly expressed in most prostate cancer cells and is an ideal target for therapy. A recent U.S. clinical study testing human imaging results of a humanized PSMA minibody (IAB2M) conjugated to IRDye800CW-NHS ester fluorescent dye was able to detect PSMA-positive primary tumors, tumor-infiltrating lymph nodes (64% sensitivity and specificity), and metastatic lesions (100% sensitivity and 65% specificity) by intraoperative fluorescent imaging in 23 patients, improving the thoroughness of tumor resection (66).

In addition, the accuracy of fluorescence imaging is closely related to the imaging device (67). In 2005, the Novadaq SPY system became the first ICG imaging system approved for use by the FDA, and since then new systems have continued to be developed and introduced (54). A variety of high-definition laparoscopic and full-color surgical cameras are now commercially available on the market, and multispectral imaging devices that recognize multiple fluorescence emission wavelengths have also been introduced (68). In addition, some systems utilize hybrid modalities combining fluorescence imaging and radiotracing (69). The application of these advanced imaging devices may further improve the accuracy and visualization of fluorescence-guided procedures.

### 2.1 Applications of fluorescence imaging

We mainly focus on the application of fluorescence imaging in the lymphatic system. It is relatively difficult to localize lymph nodes during surgery due to the complex connections between lymphatic vessels, whereas fluorescence imaging can successfully show the draining lymph nodes, and precise intraoperative resection of the associated lymph nodes can significantly improve the patient’s prognosis (70, 71). Huang et al. demonstrated that, in ICG-guided laparoscopic lymph node dissection for gastric cancer, the ICG group detected 50.5 ± 15.9 lymph nodes compared to 42.0 ± 10.3 in the control group. ICG fluorescence imaging can effectively improve the prognosis of D2 lymph node dissection in gastric cancer (72). In esophageal cancer surgery, the mean number of regional lymph nodes dissected in 84 patients under ICG guidance was 25.68 ± 12.00, with stained images of the draining lymph nodes clearly shown endoscopically (73). In prostatectomy, ICG fluorescence imaging allows real-time visualization of draining lymph nodes, successfully displaying internal iliac, external iliac, and obturator nodes (74, 75). In SLN detection of bladder cancer, fluorescence imaging successfully displays lymphatic pathways along the inferior vesical vessels to the internal iliac lymph nodes for bladder drainage detection (76). Overall, the evidence level for fluorescence imaging-guided lymph node dissection is favorable.

## 3 Status of PLND

Prognostic factors affecting patients with bladder cancer include pT, pN, lymphovascular invasion, surgical margin status, and adjuvant chemotherapy, among which pT and pN are the most important predictors of survival (77, 78). Ku et al. demonstrated that lymph node invasion was present in as high as 25% of the patients, which is one of the major risk factors for poor prognosis (79). Zehnder et al. showed that the incidence of lymph node invasion in bladder cancer ranged from 16%-35% and that the five-year recurrence-free survival and overall survival of patients with positive lymph node pathology (21%-42% and 25%-38%) were significantly worse than those of patients with negative lymph node pathology (56%-78% and 49%-69%) (15). The results of the studies of Bruins et al. and Bi et al. showed that patients with the practice of PLND had better survival outcomes than patients without PLND (80, 81). For prostate cancer, lymph node invasion is likewise a key predictor of disease progression, with a five-year recurrence-free survival rate of 50% for pN1 patients compared to 85% for pN0 patients (82). PLND is essential in intermediate- and high-risk prostate cancer.

Histologic evaluation of PLND specimens provides information on patient prognosis, with the number of metastatic lymph nodes reflecting the severity of disease progression; the more metastatic lymph nodes, the worse the patient’s prognosis. Lymph node density (number of positive nodes/number of nodes removed) has been used previously to reflect disease status, extent, and quality of surgery (83). Kassouf et al. reported five-year tumor-specific survival rates of 15.3% and 54.6% for patients with lymph node densities of >20% and <20%, respectively, which were significantly different (84). However, the number of lymph node dissection is not only affected by the number of metastatic lymph nodes and the quality of surgery, but also by a variety of other factors, such as individual differences, surgical technique, submission method, and pathologic processing methods (85). Davies et al. performed super-expanded lymph node dissection on 26 cadavers and showed that lymph node counts in the same dissection template ranged from 10-53 (86). Chen et al. conducted an autopsy on 30 autopsies to verify the clinical significance of PLND in patients with prostate cancer and similarly found significant individual differences in lymph node counts (87).

Lymph node metastasis usually occurs in the fossa occulta, but can also occur in the region of the abdominal aorta and inferior vena cava. The optimal extent of PLND remains controversial due to the inconsistency and bias of current research findings. The terms “limited PLND, standard PLND, extended PLND (ePLND) and super-extended PLND” are commonly used to denote different degrees of PLND, but the boundaries of the lymph node dissection template are not clearly defined, which makes it difficult to compare the results of different studies. While it is generally accepted that only the obturator lymph nodes are resected is referred to as limited PLND, other studies have suggested that limited PLND includes bilateral obturator, internal iliac, and external iliac lymph nodes (88). To avoid controversy, some studies have advocated the use of “level I, II, and III dissections” as an alternative to different degrees of lymph node dissection (89, 90). level I dissection includes pelvic lymph nodes below the bifurcation of the common iliac artery, mainly internal, external iliac, and obturator lymph nodes; level II dissection includes lymph nodes below the bifurcation of the aorta, including common iliac and presacral lymph nodes; and level III dissection includes para-aortic, para-inferior, and intercavernous lymph nodes upward to the origin of the inferior mesenteric artery.

Currently, PLND is the most precise staging procedure for determining lymph node metastasis, effectively preventing understaging that may occur due to the inability to detect microscopic lesions on preoperative imaging (91, 92). ePLND provides a more accurate assessment of tumor spread to the lymphatic system by examining and resecting more lymph nodes. If cancerous infiltration of lymph nodes is detected during ePLND, patients may require more aggressive treatments, such as adjuvant radiotherapy or chemotherapy. Therefore, ePLND plays a pivotal role in the diagnostic and prognostic assessment of prostate and bladder cancer (93, 94). Leissner et al. described in detail the location of metastatic lymph nodes in bladder cancer and reported the results of a prospective multicenter study at the level of level III dissection, in which metastatic lymph nodes were present in 81 out of 290 patients (27.9%), with the most common metastatic area located in the obturator fossa (25.8%), followed by the common iliac (19.0%), external iliac (15.8%), and presacral regions (8.2%), and 10% of the patients with pN1 are misdiagnosed as pN0 by level I dissection (95). The results of a randomized controlled trial by Gschwend et al. that included 401 patients comparing level I and level III dissection showed that level III dissection did not reduce the rate of tumor recurrence (88). The current consensus is that level I dissection is insufficient to remove all metastatic lymph nodes (96). Outcomes for prostate cancer are similar to those for bladder cancer, with at least 10% of metastatic lymph nodes missed by level I dissection including the obturator, internal iliac, and external iliac lymph nodes, and the oncologic outcomes of ePLND have not been shown to have a clear advantage (97–99). A study by Lestingi et al. comparing limited PLND and ePLND in 291 patients with intermediate- to high-risk prostate cancer showed no significant difference between the two in terms of biochemical recurrence-free survival, bone metastases, and mortality (100). A multi-institutional study emphasized the controversial therapeutic role of PLND, and a systematic review showed no oncologic benefit (97, 101).

Studies have shown that the extent of PLND is strongly associated with complications (91, 102, 103). Briganti et al. demonstrated that ePLND is associated with a higher incidence of lymphocele (91, 104). A systematic review and meta-analysis assessed the perioperative morbidity of PLND, with 1.8% of patients experiencing intraoperative complications such as rectal, ureteral, obturator nerve, internal and external iliac vascular injuries, and intraoperative bleeds. Postoperative complications were seen in 14.1% of patients, with lymphocele being the most common (105). Limited PLND was associated with a lower risk of complications compared to ePLND. To address the complications of ePLND and reduce negative lymph node dissection, Martini et al. used predictive modeling to omit contralateral PLND in a specific patient population (106). This model requires further external validation. Therefore, although greater PLND may improve lymph node staging, its harms and benefits need to be considered together. Surgeons should cautiously decide on the extent of PLND based on metastatic risk and clinical experience.

Overall, PLND is essential for bladder and prostate cancers at risk of lymph node metastasis. It provides a basis for staging and predicts prognosis by removing lymph nodes. The therapeutic role of PLND remains controversial. Limited PLND may miss metastatic lymph nodes, but there is no consensus on whether more extensive dissection improves survival benefits.

### 3.1 Problems with PLND

First, PLND removes lymph nodes in both tumor-associated drainage areas and non-associated drainage areas in a non-specific manner, resulting in a significant portion of unaffected lymphatic tissue being removed out of an abundance of caution (107). Second, ePLND includes bilateral obturator, internal iliac, external iliac, presacral, common iliac, and lower abdominal lymph nodes, causing extensive disruption of pelvic lymphatic anatomy. Lymphocele and lymphedema are the most common complications of PLND (108). Third, PLND does not take into account individual patient differences, and the site of lymph node metastasis can change, such as the spread of the presacral region (109, 110). To address the problem of PLND, SLN technology was introduced to target the resection of tumor-related lymph nodes by fluorescence imaging. This may reduce the number of unnecessary lymph nodes to be cleared and decrease the complication rate while detecting more metastatic lymph nodes.

## 4 Fluorescence imaging-guided SLN resection

The SLN is the first group of lymph nodes into which the metastatic tumor cells enter. Identifying and removing the first group of draining lymph nodes allows for accurate tumor staging. The results of a PLND study based on SLN targeting showed that 35% of prostate-associated lymphatic drainage was located outside the ePLND (94).

Cabanas et al. first introduced the concept of sentinel lymph node biopsy in 1977 (111). SLN resection is now a routine screening technique in breast cancer, penile cancer, and melanoma. However, in the field of prostate and bladder cancers, the SLN technique is still in the experimental stage, and the continuous advancement of new tracers, imaging modalities, and surgical techniques may increase its potential for application. Grivas et al. conducted a controlled study of patients with prostate cancer who underwent SLN resection combined with ePLND and those who underwent ePLND only, and the results showed that 5-year biochemical recurrence-free survival rate of the patients who underwent SLN resection was significantly higher than those of the control group (80.5% vs. 69.9%, P=0.03), and the 5-year biochemical recurrence-free survival rate was increased by 29.4% in the subgroup that had more than 14 lymph nodes removed (112). This demonstrates that SLN-oriented lymph node dissection may have a positive impact on tumor outcome.

### 4.1 Prostate cancer

ePLND remains the gold standard for lymph node staging of prostate cancer (113). Patients with >5% risk of lymph node metastasis as predicted by nomograms are required to undergo ePLND (114). However, ePLND is accompanied by a series of complications, such as lymphocysts, occlusive nerve injury, and lymphedema (97, 105). In addition, the poor oncologic outcome of PLND may be related to lymph node metastasis occurring outside the PLND area. Fluorescence-guided SLN resection is expected to improve this situation.

In 2011, Inoue et al. first described the use of ICG to visualize the lymphatic system during open prostatectomy (74). The study by Claps et al. demonstrated that ICG-guided lymph node dissection combined with ePLND resulted in superior staging outcomes than ePLND alone, with a higher positivity rate of fluorescence-guided dissection of lymph nodes (65.9% vs. 34.1%, P=0.01), and a higher 5-year biochemical recurrence-free survival rate in pN+ patients (54.1% vs. 24.9%, P=0.023) (115). This confirms the feasibility of fluorescence-guided lymph node dissection. In a study by Jeschke et al., that included 582 lymph nodes in 26 patients with prostate cancer, fluorescence guidance revealed an additional 120 lymph nodes (20%) compared to radiation-guided lymph node dissection (116). Subsequently, using a combination of preoperative lymphoscintigraphy and intraoperative fluorescence-guided imaging, Hruby et al. showed fluorescent staining in 75% of 38 prostate cancer patients within the expanded PLND template, with 17.5% of fluorescent lymph nodes and 16.7% of metastatic lymph nodes located outside the expanded PLND template (117). Fluorescence guidance allows complete removal of the prostatic lymphatic drainage system, with fewer lymph nodes removed compared to the PLND template, and higher diagnostic accuracy. Fluorescence-guided imaging may help physicians to accurately identify lymph nodes intraoperatively and improve the accuracy of tumor staging.

ICG has become the most commonly used fluorescent dye for visualizing lymph nodes because of its good staining effect and high contrast. However, it also has some disadvantages, free ICG forms complexes with natural proteins such as human serum albumin and rapidly migrates through tissues and the lymphatic system. This means that ICG is less specific for SLN and stains a wider range. In terms of injection methods, the most common method is the transrectal or transperineal route, where an ultrasound-guided tracer is injected into each lobe of the prostate. Manny et al.’s study evaluated different routes of administration, concluding that robotically guided intraoperative ICG injections were more effective than the cystoscopic or transrectal routes (118). De Korne et al.’s study explored the relationship between the location of tracer injections, the location of primary tumors, and the visualization of SLN association and showed that tracer injection in the paraneoplastic and intracancerous areas was more favorable for detecting metastatic lymph nodes (119). The most effective tracer injection protocol has not yet been determined, and more relevant studies are needed.

### 4.2 Bladder cancer

Fluorescence-guided techniques in bladder cancer have great potential. Its application is more difficult due to the complex anatomy of the bladder lymphatic drainage system and the complicated mechanisms of lymph node metastasis (120). Lymphatic drainage from the bladder flows from the bladder to multiple lymph nodes in the pelvis, and the SLN may extend beyond the peripheral bladder region. Leadbetter et al. summarized six pathways of normal bladder lymphatic vessels that flow into the peripheral bladder network: (1) the intramural vesical plexus (2) the insertion of lymph nodes (3) the pelvic collecting trunk (4) local pelvic nodes (comprising the external iliac, obturator, and presacral nodes) (5) lymphatics from local pelvic nodes and (6) common iliac nodes (121). In addition, in the presence of multiple tumors in the bladder, it is difficult to determine the correct drainage path for each bladder region, and there may be a crossover of drainage to the contralateral lymph nodes. Polom et al. noted that crossover was present in 45% of cases, which confirms the necessity of bilateral lymph node dissection for muscle-invasive bladder cancer (122).

In 2001, Sherif et al. published the first study of visualized SLN in patients with bladder cancer, where lymphatic visualization was performed by preoperative injection of radiotracer and blue dye around the tumor, and SLN was detected in 11 out of 13 patients (85%) (123). In 2013, Inoue et al. described for the first time the use of ICG for lymphangiography during cystectomy, and the lymphatic pathways were successfully observed in 7 out of 12 patients (76). Unlike blue dyes such as methylene blue, ICG visualizes for a long time during SLN examination by binding to macromolecules in the lymphatic vessels. However, the current problem is that ICG is not yet specific for the identification of metastatic lymph nodes. Liedberg et al. reported a false-negative rate of 19% for the identification of SLN by ICG in 75 patients, which they attributed to the diversion of afferent lymphatic drainage due to complete obstruction of the pathologic SLN by the cancer cells, which prevents the visualization of metastatic lymph nodes (124). There are few studies on specific visualization of metastatic lymph nodes in bladder cancer, and further exploration of fluorescence-targeted probes for visualization of metastatic lymph nodes is needed in the future.

In terms of factors influencing the fluorescence effect, Knapp et al. found that intravesical pressure in the bladder space was a key parameter for fluorescence visualization of lymph nodes in an animal model study of the bladder (125). Similar results were found in a human study by Shaafsma et al. in which ICG was injected with human serum albumin around the bladder tumors and the intravesical pressure was increased by filling the bladder for 15 minutes, while the control group was not filled (126). The results suggested that only 1 patient (33%) showed fluorescent SLN when the bladder was unfilled, whereas 11 out of 12 patients (92%) showed fluorescent SLN when the bladder was filled. In addition, the effect of fluorescence varied with different sites of administration, with fluorescent lymph nodes observed with submucosal injections, and not observed with plasma membrane administration. In contrast, the injection of fluorescent dye into the tumor was unable to visualize lymph nodes due to the lack of lymphatic drainage channels. Manny et al. demonstrated for the first time fluorescence-guided SLN resection in robotic-assisted surgery, and 90% of the patients were able to visualize the draining nodes, only in patients with thick bladder walls (>2 cm), where submucosal and superficial injection of ICG into the peri-tumor periphery and peristomal muscle could lead to labeling failure, implying the technique’s for thick-walled bladders limitations (127). Does preoperative neoadjuvant chemotherapy affect fluorescence-guided SLN resection? A study by Rosenblatt et al. noted that SLN detection is completely feasible in patients with muscle-invasive bladder cancer after receiving neoadjuvant chemotherapy regardless of the pT stage (128). A study by Alvaeus et al. found that the number of SLNs was significantly lower in patients with muscle-invasive bladder cancer than in patients with progressive disease after receiving neoadjuvant chemotherapy (129).

## 5 Other lymph node imaging techniques

Computed tomography (CT) and magnetic resonance imaging (MRI) are the most commonly used imaging methods for diagnosis and staging of malignant tumors. CT assessment of lymph node metastasis relies mainly on lymph node size, shape, and internal structural changes. However, metastases may be present in normal-sized lymph nodes, and enlarged lymph nodes may also be infectious or inflammatory (130, 131). Most studies have concluded that CT has a sensitivity of 30%-75% and a specificity of 56%-100% for the diagnosis of lymph node metastasis (132). Similar to CT, MRI also assesses lymph node size and morphological changes by tomographic dissection. The difference is that MRI has a better resolution of soft tissues than CT, allowing more detailed visualization of the lymph node margins and whether the interior contains fatty structures, etc.

Preclinical studies are currently developing a variety of novel MRI contrast agents, such as superparamagnetic nanoparticles, transition metal manganese-containing compounds, and superparamagnetic ionic compounds, for detecting lymph node metastasis (133–135). Superparamagnetic iron oxide nanoparticles (USPIO) are a representative class of agents used for MRI lymph node imaging (136). USPIO is lymphophilic and is phagocytosed by macrophages after intravenous injection and accumulates in lymph nodes over a long time. Normal lymph nodes contain a large number of macrophages that phagocytose USPIO, and the signal is significantly reduced on T2WI images. In metastatic lymph nodes, macrophages are replaced by tumor cells, and the reduction in T2WI signal is insignificant or non-decreasing, and benign and malignant lymph nodes can be differentiated by this feature (137). Intraoperatively, the accumulation of USPIO in the tissue can be detected using a handheld magnetometer (A special device, which is Conformité Européenne (CE) certified as a class IIa medical device) (138, 139). A prospective study in Europe reported a 100% SLN detection rate in 50 patients with intermediate- to high-risk prostate cancer, with a sensitivity and specificity of 100% and 97%, respectively, and only 3% of metastatic lymph nodes were not detected because they were outside the expanded PLND template (140). The study also noted that the application of the procedure was limited to a small part of the developed world because of the reliance on radioisotopes and nuclear medicine facilities. Another European study concluded that intraoperative magnetically guided SLN resection in patients with a 5%-20% risk of lymph node invasion could avoid ePLND because SLN resection is sufficient to detect almost all lymph node metastases (141). However, the long scanning time due to the need for patients to undergo two MRIs (pre- and post-injection) and the fact that iron oxide-based contrast agents are not readily available and not approved for use in most countries, limit the clinical application of this procedure (142).

Radionuclide imaging provides images of lymph nodes by detecting gamma rays emitted by radionuclides such as ^68^Ga, ^99m^Tc, and ^131^I (143–145). The most commonly used radionuclide is ^99m^Tc, with a half-life of 6 hours. Its labeled colloid has a diameter of 10nm-200nm, which facilitates rapid passage through the lymphatic vessels and retention in the lymph nodes (146, 147). Currently, commonly used nuclide markers include ^99m^Tc-sulfur-colloids and ^99m^Tc-Dextrans. After injection, SLN can be identified intraoperatively by the operator using a hand-held γ-detector. The advantages of radiotracer visualization of lymph nodes include good tissue penetration for detection of deep lymph nodes, preoperative radiological examination to obtain information about lymph nodes, and intraoperative radiation-guided excision of SLN. The disadvantages of this method are mainly the risk of radiation exposure, and the surgeon needs to take protective measures to complete the surgery. In addition, the risk of allergic reactions needs to be evaluated before application (148).

With the increasing importance of PSMA in the diagnosis of prostate cancer, positron emission computed tomography (PET) combined with PSMA has become a new technique for the evaluation of SLN in prostate cancer. Klingenberg et al. detected 31.4% of metastatic lymph nodes in 691 patients with prostate cancer using ^68^Ga-PSMA PET/CT (149). Sprute et al. detected metastatic lymph nodes using the ^18^F-PSMA-1007 tracer, and 84 out of 92 positive lymph nodes were determined to be true positives by pathology (150). A meta-analysis including 10 studies using ^68^Ga-PSMA PET/CT to assess lymph node metastasis showed a combined sensitivity of 61% and specificity of 96% per patient. For patients at low risk of lymph node metastasis, PSMA PET/CT-negative patients may reduce unnecessary lymph node dissection procedures (151). More relevant prospective large-scale cohort studies are needed to prove its accuracy.

## 6 Combined fluorescence and radionuclide imaging to guide PLND

Fluorescence imaging is less time-consuming, easy to perform, safe, and convenient. The disadvantage is that fluorescence signal penetration is limited, making it difficult to detect deep lesions. In contrast, γ-rays hardly attenuate in tissues, and radionuclide-labeled fluorescent substances can provide in-depth lymph node information by single-photon emission computed tomography (SPECT) or PET examination (152). Combining fluorescence imaging and radionuclide imaging can effectively solve the problem of insufficient fluorescence penetration (153).

In prostate cancer, fluorescence combined with radionuclide imaging or biomarker PSMA aims to improve the accuracy of identifying lymph node metastases, avoiding the omission of potentially metastatic lymph nodes, and reducing complications associated with ePLND. ICG-^99m^Tc nanocolloids were the first hybrid tracer used to improve lymph node dissection procedures (154). Labeling methods using hybridization of ^99m^Tc and ICG reduce their respective limitations and offer the advantages of both. Van der Poel et al. injected a hybrid tracer into the prostate, which allowed for preoperative SLN localization and intraoperative detection of resection (155). Jeschke et al. described an alternative approach with sequential injection of the radiocolloid ^99m^Tc and free ICG, which also allowed for preoperative and intraoperative lymph node detection (116). Wit et al. compared the two methods and showed that the hybridization approach (ICG-^99m^Tc) appeared to be more effective than the sequential use of ^99m^Tc nanocolloid and ICG, with a higher incidence of metastatic fluorescent lymph nodes in the hybridization group (7.4%) than in the sequential group (2.6%; p = 0.002) (156). Hinsenveld et al. evaluated the use of fluorescence imaging in combination with PSMA PET/CT, injecting ICG-^99m^Tc nanocolloids or sequentially applying ^99m^Tc and free ICG in patients who had a negative preoperative PSMA PET/CT, and accurate lymph node staging was possible in 50 out of 53 patients (94% diagnostic accuracy), and all pN+ patients were correctly identified (157).

Radionuclide imaging in bladder cancer can accurately determine the location of lymph nodes, but cannot identify tiny lymphatic vessels. In contrast, fluorescence imaging can accurately observe the flow route of lymphatic fluid in lymphatic vessels. Therefore, the combination of fluorescence and radiotracer shows good application prospects. Studies have demonstrated that the combination of fluorescence and radiotracer can detect more than 80% of the patient’s anterior region, including the SLN outside the PLND template. In 2022, Rietbergen et al. performed the first study of SLN resection for bladder cancer using ICG-^99m^Tc nanocolloid and demonstrated that 83% of SLNs detected by preoperative imaging could be used for intraoperative SLN resection, which was safe and feasible in three patients whose SLNs were located outside of the ePLND (158).

## 7 Multimodal imaging

The simultaneous use of two or more imaging modalities has triggered the emergence of the concept of multimodal imaging for better characterization of lymphatic vessels and lymph nodes (159, 160). Imaging techniques such as CT, MRI, SPECT, and PET, in combination with fluorescence imaging, have been used for lymph node identification and real-time image-guided resection in preclinical applications (161–164). In addition, the combination of imaging techniques with tumor biomarkers such as PSMA helps to achieve accurate detection of metastatic lymph nodes. Discussions on the biological mechanisms of tumorigenesis, progression, metastasis, and heterogeneity have provided a basis for selecting appropriate biomarkers as targeted imaging drugs (165–167). Nevertheless, drugs ultimately need to be rigorously evaluated to ensure safety and feasibility in the human body. The evolving variety of imaging modalities has collectively contributed to the advancement of multimodal imaging, providing a more precise and reliable solution to the problem of lymph node metastasis in prostate and bladder cancer (163, 168).

## 8 Limitations and future perspectives of fluorescence imaging

Fluorescence imaging has great potential for lymph node detection in prostate and bladder cancer, providing an easy method to recognize and identify lymph nodes (169, 170). However, its accuracy still needs to be combined with the operator’s clinical experience and preoperative examination. Currently, postoperative pathology is still the gold standard for determining lymph node metastasis, and fluorescence imaging has not yet been able to achieve intraoperative identification of metastatic lymph nodes (171). Currently, it is difficult to directly compare the results of clinical studies from different research centers for reasons that include the lack of consensus on the assessment of clinical outcomes of fluorescence imaging, as well as the inconsistency of various parameters, observer differences, and the learning curve of the technique (172). The development and application of novel fluorescent probes are challenged by the variety of parameters that affect the effectiveness of fluorescent imaging (173). Continued development and precise matching of imaging systems and fluorescent contrast agents may further improve detection.

In addition, understanding the effects of different routes of administration of fluorescent contrast agents, such as intravenous, intratumor, paratumor, subcutaneous, and intradermal injections, on the imaging effect is also extremely important for reducing drug toxicity, decreasing the incidence of adverse reactions and complications, and thus facilitating their clinical translation (174). Fluorescence imaging-guided lymph node dissection in patients with prostate and bladder cancer is still in its infancy, and the goal of clinical studies is to evaluate the safety, feasibility, and optimal dose of fluorescent contrast agents. In the future, multimodal imaging combining multiple imaging modalities and tumor markers may allow for a more comprehensive evaluation of pelvic lymph nodes.

## 9 Conclusion

For prostate and bladder cancers, pelvic lymphatic drainage is complex and variable, making metastatic lymph nodes easily missed. Determining the number and extent of PLND is a challenge for urologists. Fluorescence-guided SLN resection is an important tool to solve this challenge and contributes to accurate tumor staging. Due to the limited penetration depth of fluorescence imaging, radionuclide imaging compensates for this deficiency. The combined fluorescence and radionuclide-guided approach allows for more accurate lymph node staging. The concept of multimodal imaging brings new hope for identifying pelvic metastatic lymph nodes. In the future, an expert consensus on the results of fluorescence imaging of lymph nodes should be formulated as soon as possible, and comprehensive evaluation through high-quality clinical studies is needed.
